# *MBL2 *and Hepatitis C Virus Infection among Injection Drug Users

**DOI:** 10.1186/1471-2334-8-57

**Published:** 2008-05-01

**Authors:** Elizabeth E Brown, Mingdong Zhang, Rebecca Zarin-Pass, Toralf Bernig, Fan-Chen Tseng, Nianqing Xiao, Meredith Yeager, Brian R Edlin, Stephen J Chanock, Thomas R O'Brien

**Affiliations:** 1 Division of Cancer Epidemiology and Genetics, Center for Cancer Research, National Cancer Institute, National Institutes of Health, Bethesda, Maryland, USA; 2 Center for Cancer Research, National Cancer Institute, National Institutes of Health, Department of Health and Human Services, Bethesda, Maryland, USA; 3 SAIC-Frederick, Inc., Core Genotyping Facility, Advanced Technology Center, National Cancer Institute, Gaithersburg, Maryland, USA; 4 Center for the Study of Hepatitis C, Weill Medical College of Cornell University, New York, New York, USA; 5 Urban Health Study, University of California, San Francisco, USA

## Abstract

**Background:**

Genetic variations in *MBL2 *that reduce circulating levels and alter functional properties of the mannose binding lectin (MBL) have been associated with many autoimmune and infectious diseases. We examined whether *MBL2 *variants influence the outcome of hepatitis C virus (HCV) infection.

**Methods:**

Participants were enrolled in the Urban Health Study of San Francisco Bay area injection drug users (IDU) during 1998 through 2000. Study subjects who had a positive test for HCV antibody were eligible for the current study. Participants who were positive for HCV RNA were frequency matched to those who were negative for HCV RNA on the basis of ethnicity and duration of IDU. Genotyping was performed for 15 single nucleotide polymorphisms in *MBL2*. Statistical analyses of European American and African American participants were conducted separately.

**Results:**

The analysis included 198 study subjects who were positive for HCV antibody, but negative for HCV RNA, and 654 IDUs who were positive for both antibody and virus. There was no significant association between any of the genetic variants that cause MBL deficiency and the presence of HCV RNA. Unexpectedly, the *MBL2 *-289X promoter genotype, which causes MBL deficiency, was over-represented among European Americans who were HCV RNA negative (OR = 1.65, 95% CI 1.05–2.58), although not among the African Americans.

**Conclusion:**

This study found no association between genetic variants that cause MBL deficiency and the presence of HCV RNA. The observation that *MBL2 *-289X was associated with the absence of HCV RNA in European Americans requires validation.

## Background

Mannose binding lectin (MBL) is an acute phase reactant that is secreted from the liver and is critical in host defenses against a spectrum of bacterial, fungal, viral, and parasitic pathogens. MBL directly mediates opsonophagocytosis and acvation of the C-type lectin complement pathway by binding microbial mannose and N-acetylglucosamine surface residues. MBL deficiency has been associated with a range of auto-immune and infectious diseases, including HIV-1 and hepatitis B viral infections [1-4].

Low circulating levels of MBL have been associated with common genetic variants in the *MBL2 *gene, which is the only functional human gene for MBL. *MBL2 *is located on the long arm of chromosome 10 (10q11.2-11) and consists of four exons [5]. Exon 1 includes three nonsynonymous single nucleotide polymorphisms (SNPs), known as D, B and C alleles, that alter the function as well as circulating levels of MBL [6,7]. These SNPs, in combination with two proximal promoter variants (-618H/L and -289Y/X) [8,9] and a variant in the 5' untranslated region (UTR) of MBL2 (-65Q/P) [6], form seven well-characterized 'secretor' haplotypes that alter functional activity, namely, opsonophagocytosis, complement-activation and circulating levels of MBL [10].

In the United States, chronic hepatitis C virus (HCV) infection is a leading cause of cirrhosis, end-stage liver disease and hepatocellular carcinoma. The medical literature on MBL and HCV infection is limited. Most previous studies focus on the relationship between *MBL2 *variants and either response to antiviral treatment or the risk of developing fibrosis/cirrhosis [11]. In the present study, we evaluated whether common variations in *MBL2 *influence the spontaneous resolution of HCV infection in a population of injection drug users (IDU).

## Methods

### Study population

The present analysis is part of a comprehensive evaluation of the relationship between human genetics and HCV infection among IDUs enrolled in the Urban Health Study (UHS). As previously described [12], participants enrolled in UHS were recruited from street settings in six inner-city San Francisco Bay area neighborhoods. All persons 18 years of age or older were eligible for enrollment if they had injected drugs within the past 30 days or previously participated in the Urban Health Study. New participants were screened for visible signs of recent or chronic injection (i.e., recent venipuncture sites or scars). After the participant provided written informed consent, a blood sample and responses to a questionnaire were collected. Study procedures were approved by Committee on Human Subjects Research at University of California, San Francisco and the Institutional Review Board of the National Cancer Institute.

Participants enrolled in the UHS from 1998 through 2000 who had a positive test for HCV antibody were eligible for the current study, which is a case-control study based on cross-sectional data. These participants were tested for HCV RNA. Those positive for HCV RNA were frequency matched (maximum 4:1 ratio) to those who were negative for HCV RNA on the basis of self-reported ethnic background and duration of IDU (± 5 years). Because IDUs had very limited access to anti-HCV treatment at the time of subject recruitment [13,14], it is highly likely that the HCV RNA negative subjects in this study had recovered spontaneously. Participants who were positive for HBV surface antigen (HBsAg) were later excluded from the analyses because of the complex relationship between HCV infection and HBV infection in this population [24]. The subjects of this analysis consisted of 198 HCV RNA negative participants (109 European Americans and 89 African Americans) and 654 HCV RNA positive participants (344 European Americans and 310 African Americans).

### Laboratory methods

#### Viral assays

We detected HCV antibodies using the HCV EIA version 3 (Ortho Diagnostics, Raritan, NJ), HCV RNA by a branched-chain DNA assay (HCV bDNA version 3.0, Bayer, Tarrytown, NY; analytic sensitivity, 2.5 × 10^3 ^copies/ml) and HBsAg with the Genetic Systems HBsAg EIA (Bio-Rad, Redmond, WA). We tested for antibodies to HIV-1 infection in plasma using the Genetic Systems rLAV EIA assay (Bio-Rad, Redmond, WA). Reactive samples were confirmed by HIV-1 Western Blot (Cambridge Biotech, Worcester, MA).

#### Genotyping

Based on recent resequencing of *MBL2*, we selected fifteen SNPs from exonic, intronic and promoter regions of *MBL2 *to include loci that were previously associated with circulating MBL levels and to capture haplotype diversity in both the 5' and 3' blocks (Table 1) [15,16]. Information on all assays is publicly available (including primers, probes and conditions) on the SNP500 Cancer website [17,18]. Details of the nomenclature used to describe these sequence variations are also available [19,20]. We extracted genomic DNA from cryopreserved lymphocytes using a modified salt precipitation-extraction method (Gentra Systems, Minneapolis, MN) or from granulocytes using a silica membrane binding method (Qiagen Inc., Valencia, CA). Genotyping was performed using optimized TaqMan™ assays and analyzed on the ABI 7900HT platform (ABI, Foster City, CA). We identified duplicate subjects with the AmpFLSTR Profiler Plus^® ^PCR amplification kit (PE Applied Biosystems, Foster City, CA) to type nine tetranucleotide short tandem repeat loci and the Amelogenin locus. A blinded duplicate analysis of 5% of study samples demonstrated greater than 99% concordance.

**Table 1:** Common variants of *MBL2 *genotyped in the Urban Health Study

**Variant**	**dbSNP Identifier**	Secretor*	**Amino Acid Change**	**Function**
T-1964C	rs1031101			
G-618C	rs11003125	H/L (aka -550)		H = high, L = low
G-289C	rs7096206	Y/X (aka -221)		Y = high, X = low
T-65C	rs7095891	Q/P (aka +4)		
Ex1 C-34T	rs5030737	A/D (Codon 52)	R52C	D = low
Ex1 G-27A	rs1800450	A/B (Codon 54)	G54D	B = low
Ex1 G-18A	rs1800451	A/C (Codon 57)	G57E	C = low
IVS2 G-630A	rs4935046			
IVS2 T-250C	rs1838066			
IVS3 G-28C	rs930508			
Ex4 C+5G	rs930507			
Ex4 T-1483C	rs10082466			
Ex4 G-1067A	rs10824792			
Ex4 G-901A	rs2120132			
Ex4 G-710A	rs2099902			

**NOTE: **Six common variants form secretor haplotypes including -618, -289, -65, Ex1 -34, Ex1 -27, Ex1 – 18. Nomenclature for description of sequence variations is described in

#### Statistical analysis

All analyses were conducted separately among European American and African American participants. We first examined departure from Hardy-Weinberg proportions for each locus among the participants that were positive for HCV RNA and then evaluated differences in genotype frequencies by HCV RNA status. We estimated the relative risk of being HCV RNA negative using the prevalence odds ratio (OR) and corresponding 95% confidence interval (CI) calculated with logistic regression adjusted for sex, duration of IDU and HIV-serostatus. Consistent with some previous studies of HCV outcome, we examined odds ratios for being HCV negative because it is the rarer outcome. We did not include age in the regression models because in UHS subjects this variable is highly correlated with duration of IDU. The Pearson χ^2 ^test was used to calculate p-values; p 0.05 (two-tailed) was considered statistically significant.

The seven classical secretor haplotypes (HYPA, LYPA, LYQA, LXPA, HYPD, LYPB and LYQC; Table 1) were analyzed based on prior studies that demonstrated associations with circulating levels of MBL. Overall heterogeneity (global score test) and individual secretor haplotype frequencies, adjusted for covariates (sex, duration of IDU and HIV-1 infection), were estimated by the expectation-maximization progressive insertion algorithm (HaploStats version 1.1.0), as previously described [21]. This method is based on empirical distributions of a minimum of 10,000 and maximum of 50,000 permutations. We also examined diplotypes formed by the Y/X promoter variant and the three common structural variants from exon 1 [10,16]. The wild-type structural allele is designated as "A" and the nonsynonymous SNPs (D, B and C) are designated as "O".

## Results

Characteristics of the study population are described in Table 2. Of the 453 European American participants, those who were HCV RNA negative did not notably differ from those who were HCV RNA positive with regard to age (median: 43 years in each group) or duration of IDU (median: cleared 23 years; chronic, 24 years). Male gender, however, was more common among HCV RNA positive participants (71.8%) compared to those who were HCV RNA negative (62.4%; p = 0.06). In addition, HIV-1 infection was more common among European American participants who were HCV RNA positive (11.9%) compared to those who were HCV RNA negative (4.6%; p = 0.03).

**Table 2:** General demographic characteristics of the Urban Health Study population genotyped for common variants in *MBL2*

	***European Americans***			***African Americans***		
		
	**HCV RNA Negative (n = 109)**	**HCV RNA Positive (n = 344)**	**P**	**HCV RNA Negative (n = 89)**	**HCV RNA Positive (n = 310)**	**P**
Gender, male (%)	68 (62.4)	247 (71.8)	0.06	54 (68.7)	192 (61.2)	0.83
Age at blood draw, median years (range)	43 (25–63)	43 (23–65)	0.46	47 (31–64)	47 (28–82)	0.16
Duration of IDU, median years (range)	23 (5–41)	24 (10–49)	0.83	26 (8–44)	27 (11–51)	0.13
HIV-1 serostatus						
Seropositive	5 (4.6)	41 (11.9)		8 (9.0)	36 (11.6)	
Seronegative	104 (95.4)	303 (88.1)	0.03	81 (91.0)	274 (88.4)	0.49

Among the 399 African Americans included in this investigation, participants who were HCV RNA negative were similar to those who were HCV RNA positive with regard to age (median: 47 years in each group), duration of IDU (median: cleared 26 years; chronic, 27 years), gender (males: chronic, 61.9%; cleared, 60.7%) and HIV-1 infection (chronic HCV infection, 11.6%; cleared, 9.0%).

### HCV RNA and MBL2 genotypes

In separate evaluations of the European American and African American participants who were positive for HCV RNA, genotypic distributions for all fifteen variants were consistent with Hardy-Weinberg proportions (*P *≥ 0.05; Table 3). Among European American participants, the -289C-containing variant (-289X) of the proximal promoter, which causes MBL deficiency, was over-represented among those who were HCV RNA negative compared to participants who were positive for HCV RNA (OR = 1.65, 95% CI 1.05–2.58). There were no notable associations with the other promoter variants that have been previously shown to alter MBL levels [G618C (H/L) or T65C (P/Q)] or with either structural locus commonly found among European Americans (D or B). Associations with other *MBL2 *variants did not achieve statistical significance.

**Table 3:** Common variants in *MBL2 *and odds ratio for being HCV RNA negative among injection drug users

		***European Americans***					***African Americans***				
			
**Locus**	**Genotype**	**HCV RNA Negative**	**%**	**HCV RNA Positive**	**%**	**OR (95% CI)**	**HCV RNA Negative**	**%**	**HCV RNA Positive**	**%**	**OR (95% CI)**
T-1964C											
	TT	83	77.6	256	76.0	1.00	82	93.2	288	94.1	1.00
	TC	23	21.5	73	21.7	0.94 (0.55–1.60)	6	6.8	17	5.6	1.27 (0.48–3.36)
	CC	1	0.9	8	2.4	0.44 (0.05–3.51)	0	0.0	1	0.3	NA
	TC + CC	24	22.4	81	24.0	0.91 (0.54–1.53)	6	6.8	18	5.9	1.20 (0.46–3.14)
G-618C (H/L)											
	CC	44	41.9	124	36.4	1.00	69	80.2	233	76.9	1.00
	CG	47	44.8	164	48.1	0.80 (0.50–1.29)	15	17.4	67	22.1	0.76 (0.41–1.42)
	GG	14	13.3	53	15.5	0.72 (0.36–1.43)	2	2.2	3	1.0	2.63 (0.42–16.43)
	CG + GG	61	58.1	217	63.6	0.80 (0.51–1.26)	17	19.8	70	23.1	0.83 (0.46–1.50)
G-289C (Y/X)											
	GG	59	55.1	218	66.3	1.00	63	71.6	218	70.6	1.00
	GC	42	39.3	103	31.3	1.56 (0.98–2.49)	23	26.1	87	28.2	0.92 (0.53–1.58)
	CC	6	5.6	8	2.4	2.72 (0.90–8.23)	2	2.3	4	1.3	2.01 (0.35–11.42)
	GC + CC	48	44.9	111	33.7	1.65 (1.05–2.58)	25	28.4	91	29.4	0.97 (0.57–1.63)
T-65C (P/Q)											
	CC	64	59.8	197	57.4	1.00	21	23.9	64	20.8	1.00
	CT	38	35.5	127	37.0	0.93 (0.59–1.48)	43	48.9	160	51.9	0.81 (0.44–1.48)
	TT	5	5.0	19	5.5	0.75 (0.27–2.09)	24	27.3	84	27.3	0.85 (0.43–1.67)
	CT + TT	43	4.7	146	42.6	0.90 (0.58–1.40)	67	76.1	244	79.2	0.83 (0.47–1.47)
Ex1 C-34T (D)											
	CC	95	87.2	300	87.5	1.00	87	97.8	303	98.1	1.00
	TC	13	11.9	42	12.2	1.06 (0.54–2.10)	2	2.3	6	1.9	1.11 (0.22–5.62)
	TT	1	0.9	1	0.3	2.71 (0.16–45.25)	0	0.0	0	0.0	NA
	TC + TT	14	12.8	43	12.5	1.00 (0.52–1.91)	2	2.3	6	1.9	1.11 (0.22–5.62)
Ex1 G-27A (B)											
	GG	85	78.0	256	75.5	1.00	83	93.3	285	93.4	1.00
	GA	23	21.1	75	22.1	0.90 (0.53–1.53)	6	6.7	19	6.2	1.10 (0.42–2.86)
	AA	1	0.9	8	2.4	0.42 (0.05–3.46)	0	0.0	1	0.3	NA
	GA + AA	24	22.0	83	24.5	0.87 (0.52–1.46)	6	6.7	20	6.6	1.04 (0.40–2.70)
Ex1 G-18A (C)											
	GG	95	92.2	312	96.0	1.00	49	56.3	168	55.6	1.00
	GA	8	7.8	13	4.0	2.26 (0.89–5.73)	29	33.3	116	38.4	0.86 (0.51–1.45)
	AA	0	0.0	0	0.0	NA	9	10.3	18	6.0	1.68 (0.71–3.99)
	GA + AA	8	7.8	13	4.0	2.26 (0.89–5.73)	38	43.7	134	44.4	0.97 (0.60–1.57)
IVS2 G-630A											
	GG	22	20.8	59	17.9	1.00	49	57.6	168	54.4	1.00
	GA	55	51.9	161	48.8	0.89 (0.50–1.59)	31	36.5	127	41.1	0.84 (0.51–1.39)
	AA	29	27.4	110	33.3	0.68 (0.36–1.30)	5	5.9	14	4.5	1.22 (0.41–3.60)
	GA + AA	84	79.3	271	82.1	0.82 (0.47–1.43)	36	42.4	141	45.6	0.87 (0.54–1.42)
IVS2 T-250C											
	TT	42	40.4	119	35.6	1.00	72	80.9	236	76.9	1.00
	TC	48	46.2	161	48.2	0.84 (0.52–1.36)	15	16.9	68	22.2	0.73 (0.39–1.36)
	CC	14	13.5	54	16.2	0.72 (0.36–1.43)	2	2.3	3	1.0	2.52 (0.40–15.75)
	TC + CC	62	59.6	215	64.4	0.83 (0.53–1.31)	17	19.1	71	23.1	0.79 (0.44–1.43)
IVS3 G-28C											
	GG	72	66.7	239	70.7	1.00	36	41.9	135	43.8	1.00
	GC	34	31.5	93	27.5	1.28 (0.79–2.07)	39	45.3	144	46.8	1.02 (0.61–1.70)
	CC	2	1.9	6	1.8	0.98 (0.19–5.00)	11	12.8	29	9.4	1.42 (0.65–3.14)
	GC +CC	36	33.3	99	29.3	1.25 (0.78–1.99)	50	58.1	173	56.2	1.08 (0.66–1.76)
Ex4 C+5G											
	GG	70	66.7	244	71.6	1.00	41	46.1	143	46.6	1.00
	GC	33	31.4	92	27.0	1.31 (0.81–2.14)	39	43.8	138	45.0	0.98 (0.60–1.62)
	CC	2	1.9	5	1.5	1.17 (0.22–6.27)	9	10.1	26	8.5	1.22 (0.53–2.82)
	GC + CC	35	33.3	97	28.4	1.32 (0.82–2.11)	48	53.9	164	53.4	1.02 (0.63–1.64)
Ex4 T-1483C											
	TT	65	60.2	193	58.5	1.00	25	28.7	88	29.6	1.00
	TC	36	33.3	117	35.5	0.91 (0.57–1.47)	32	36.8	155	52.2	0.72 (0.40–1.30)
	CC	7	6.5	20	6.1	1.13 (0.45–2.83)	30	34.5	54	18.2	2.02 (1.07–3.81)
	TC + CC	43	39.8	137	41.5	0.94 (0.60–1.47)	62	71.3	209	70.4	1.05 (0.62–1.79)
Ex4 G-1067A											
	GG	18	17.5	46	13.8	1.00	62	72.9	199	65.9	1.00
	GA	48	46.6	156	46.8	0.72 (0.38–1.37)	19	22.4	92	30.5	0.67 (0.38–1.20)
	AA	37	35.9	131	39.3	0.65 (0.33–1.26)	4	4.7	11	3.6	1.31 (0.40–4.32)
	GA +AA	85	82.5	287	86.2	0.70 (0.38–1.28)	23	27.1	103	34.1	0.73 (0.43–1.25)
Ex4 G-901A											
	AA	61	58.1	187	57.0	1.00	26	29.2	90	29.8	1.00
	GA	37	35.2	118	36.0	0.97 (0.60–1.55)	35	39.3	160	53.0	0.75 (0.42–1.33)
	GG	7	6.7	23	7.0	1.02 (0.41–2.52)	28	31.5	52	17.2	1.87 (0.99–3.53)
	GA + GG	44	41.9	141	43.0	0.69 (0.61–1.51)	63	70.8	212	70.2	1.03 (0.61–1.73)
Ex4 G-710A											
	AA	65	60.7	196	58.3	1.00	18	20.2	65	21.3	1.00
	GA	34	31.8	120	35.7	0.86 (0.54–1.39)	34	38.2	150	49.2	0.82 (0.43–1.56)
	GG	8	7.5	20	6.0	1.31 (0.55–3.17)	37	41.6	90	29.5	1.50 (0.78–2.88)
	GA + GG	42	39.3	140	41.7	0.92 (0.59–1.44)	71	79.8	240	78.7	1.07 (0.60–1.93)

**NOTE: **The most frequent homozygotes among controls served as the referent. Genotypic odds ratios were adjusted for sex, duration of injection drug use, and HIV-serostatus; OR (odds ratio); 95% CI (confidence interval); and NA, not applicable.

In contrast to our observation among European American participants, the -289C (low promoter) genotype was unrelated to HCV RNA status among African American participants (OR, 0.97; *P *= 0.94). Homozygosity for the C allele of the T-1483C exon 4 polymorphism, which is a synonymous variant without established functional effects, was associated with a two-fold increased likelihood of being HCV negative in the African Americans, but there was no overall association for 1483C carriers (OR, 1.05; 95% CI, 0.62–1.79). HCV clearance among African American participants was not significantly associated with other promoter, structural or intronic variants of *MBL2*.

### HCV RNA and haplotypes of MBL2

Among European Americans, the LXPA haplotype (the only secretor haplotype containing -289C [X]) was associated with an increased likelihood of being negative for HCV RNA when compared to the HYPA haplotype (Table 4; OR = 1.73, 95% CI 1.10–2.74; p = 0.01). LXPA is associated with lower MBL levels compared to HYPA. In an additive model, each copy of LXPA was associated with a nearly 2-fold increase in risk (P-trend = 0.01). Among African American participants, there were no significant associations of secretor haplotypes and HCV RNA.

**Table 4:** Odds ratio for risk of being HCV RNA negative among European American and African American injection drug users, by MBL2 secretor haplotype profile.

							**Haplotype frequency estimates**				
								
**Secretor Haplotype**							**Total**	**HCV RNA Negative**	**HCV RNA Positive**	**P**	**OR (95% CI)**
**European Americans**											
	-618	-289	-65	Ex1 -34	Ex1 -27	Ex1 -18					
HYPA	G	G	C	C	G	G	32.3%	29.2%	33.2%	0.24	referent
LYQA	C	G	T	C	G	G	21.2%	18.5%	22.1%	0.23	0.93 (0.58–1.51)
LXPA	C	C	C	C	G	G	19.5%	25.2%	17.7%	0.01	1.73 (1.10–2.74)
LYPB	C	G	C	C	A	G	12.9%	11.5%	13.3%	0.49	0.93 (0.53–1.64)
HYPD	G	G	C	T	G	G	6.5%	6.9%	6.4%	0.66	1.19 (0.59–2.39)
LYPA	C	G	C	C	G	G	5.1%	5.0%	5.1%	0.94	0.92(0.40–2.13)
LYQC	C	G	T	C	G	A	2.5%	3.9%	2.0%	0.08	3.27 (1.18–9.05)
*Global score statistic*										*0.14*	

**African Americans**											
LYQA	C	G	T	C	G	G	27.3%	24.8%	28.0%	0.38	referent
LYQC	C	G	T	C	G	A	25.7%	27.0%	25.3%	0.69	1.24 (0.76–2.01)
LYPA	C	G	C	C	G	G	16.5%	18.4%	15.9%	0.49	1.24(0.70–2.21)
LXPA	C	C	C	C	G	G	15.4%	15.6%	15.4%	0.89	1.23 (0.69–2.18)
HYPA	G	G	C	C	G	G	10.7%	9.8%	10.9%	0.74	1.10 (0.57–2.12)
LYPB	C	G	C	C	A	G	3.4%	3.4%	3.4%	0.91	1.15 (0.44–3.01)
HYPD	G	G	C	T	G	G	1.0%	1.1%	1.0%	0.92	1.21 (0.23–6.29)
*Global score statistic*										*0.98*	

Relative haplotype frequencies adjusted for sex, duration of injection drug use and HIV-1 serostatus.

Haplotypes appear in order of descending haplotype frequencies among the comparison group stratified by race.

P-value for LYPC among European Americans is not statistically signficant due to permutations based on small haplotype frequencies.

The global score test evaluates differences between cases and controls in the overall haplotype distribution.

We next examined the relationship between the *MBL2 *Y/X, A/0 diplotypes and HCV RNA (Table 5), comparing participants who carried YA/YA, which has been associated with the highest MBL levels, to participants with other diplotypes in descending order of previously associated serum MBL levels. Among European American participants, those with XA/YO were more likely to be HCV RNA negative (OR, 2.31; 95% CI, 1.07–5.02). Collapsing the diplotypes into four categories based on a previous report of MBL levels [15], participants in the lowest category (XA/YO and YO/YO) were more likely to be HCV RNA negative than those in the highest category (YA/YA diplotype; OR, 2.00; 95% CI, 1.05–3.82). Participants in intermediate categories were no more likely to be HCV RNA negative than those with the YA/YA diplotype. There were no notable associations of any MBL Y/X, A/0 diplotype and HCV RNA among African American participants (Table 5).

**Table 5:** *MBL2 *diplotypes and odds ratio for risk of being HCV RNA negative among injection drug users

	**European Americans**				**African Americans**			
		
**Diplotype**	**HCV RNA Negative**	**HCV RNA Positive**	**OR (95% CI)**	OR (95% CI)*	**HCV RNA Negative**	**HCV RNA Positive**	**OR (95% CI)**	OR (95% CI)*
**YA/YA**	32.1%	37.8%	1.00	1.00	30.3%	31.3%	1.00	1.00
**XA/YA**	25.7%	22.7%	1.36 (0.77–2.43)	1.36 (0.77–2.43)	18.0%	18.1%	1.01 (0.50–2.05)	1.01 (0.50–2.05)
**YA/YO**	16.5%	24.1%	0.81 (0.43–1.55)	0.99 (0.77–1.79)	29.2%	31.0%	0.96 (0.52–1.78)	1.00 (0.55–1.83)
**XA/XA**	5.5%	2.3%	2.74 (0.88–8.52)		2.3%	1.3%	2.08 (0.35–12.21)	
**XA/YO**	12.8%	7.3%	2.31 (1.07–5.02)	2.00	7.9%	10.0%	0.82 (0.33–2.08)	1.13
**YO/YO**	7.3%	5.8%	1.61 (0.65–4.03)	(1.05–3.82)	12.4%	8.4%	1.49 (0.65–3.41)	(0.57–2.24)

*OR for grouped diplotypes. Diplotypes were ordered and grouped on the basis of previously reported serum MBL levels among Caucasians [13]. Mean serum MBL [mg/l] in that report were: YA/YA, 4.78; XA/YA, 2.83; YA/YO, 1.58; XA/XA, 1.39; XA/YO, 0.19; YO/YO, 0.05.

## Discussion

In this study, *MBL2 *genotypes or haplotypes associated with MBL deficiency were not, in general, associated with the likelihood of being negative for HCV RNA. Among European Americans, the -289C (X) promoter variant, which is associated with moderately lower MBL levels, was actually more common in participants who were negative for HCV RNA than those who were positive. Among African Americans, there was no compelling evidence of HCV RNA status with any of the *MBL2 *genotypes or haplotypes evaluated. To our knowledge there are no previous studies of common variation in *MBL2 *and spontaneous clearance of HCV infection to corroborate these results [11], but the size of the study, our multiethnic population and gene coverage suggest that clearance of HCV infection is not impaired by MBL insufficiency.

The association of HCV RNA negative status with *MBL2 *-289C (X) [and LXPA, the haplotype associated with -289C] among European Americans may be due to chance. This 'protective effect' is contrary to most previous observations where MBL deficiency predisposed to a worsened course of infection. In addition, *MBL2 *structural variants were not significantly associated with HCV RNA negative status among either European Americans or African American participants. Conversely, it should be noted that the preservation of heterozygosity for *MBL2 *loci and the corresponding range of circulating MBL levels suggests that low to moderate levels of MBL could be advantageous in some circumstances [16]. In that regard, an association between genotypes that produce low MBL levels and more favorable outcome among patients with tuberculosis has been reported [22]. The association of HCV RNA negative status with *MBL2 *-289C (X) found among European Americans in the present study requires replication.

We defined HCV outcome on the basis of results from a single HCV EIA and a single HCV bDNA assay. False positive HCV EIA results are unlikely to have affected our findings in a meaningful way because the high prevalence of HCV antibodies found among IDUs combined with the high specificity of the assay we used [23] yield a positive predictive value exceeding 99%. Because this is a case-control study based on cross-sectional data, we could not use the clinical definition of HCV "clearance" that is based on the results of two HCV RNA assays at least 6 months apart. It should also be noted that that we defined a positive HCV RNA result on the basis of an assay with a detection limit of 2500 copies/ml. In another analysis based on these test results, Tseng et al [24] found that the relative risks in UHS for previously reported predictors of chronic HCV infection (i.e., older age, African ancestry, presence of HBsAg, HIV infection) were very consistent with studies of 'HCV clearance' [25]. We are confident, therefore, that our study design is able to detect true differences in HCV outcome. We do not believe that our results are confounded by gender or HIV-1 status as we controlled for these variables in logistic regression models. The number of HIV-1-infected subjects in the study was too few for a meaningful evaluation of the independent effect of the *MBL2 *variants on HIV-1 serostatus.

## Conclusion

In summary, our results suggest that HCV clearance is not among the infectious outcomes for which variants of *MBL2 *that cause MBL deficiency are deleterious. Our unexpected observation that MBL2 -289X was associated with the absence of HCV RNA in European Americans requires validation in other populations.

## Competing interests

The authors declare that they have no competing interests.

## Authors' contributions

EEB conducted data analysis and drafted the initial manuscript. MZ helped design the study. RZ-P contributed to study design and data analysis, TB performed genotyping, F-CT conceived of the analysis and helped design the study. NX performed data analysis. MY participated in genotyping and data analysis. BRE helped design the project and supervised the field work. SJC supervised genotyping and contributed to data analysis. TRO'B conceived of and coordinated the project, participated in the data analysis and revised the manuscript. All authors read and approved the final manuscript.

## Pre-publication history

The pre-publication history for this paper can be accessed here:


